# Dental Schools Abroad

**Published:** 1899-01

**Authors:** H. L. Ambler

**Affiliations:** Cleveland, Ohio.


### Dental Schools Abroad.

BY H. L. AMBLER, M.D., D.D.S., CLEVELAND, OHIO.

Read before the Ohio State Dental Society December 7th, 1898.

The Edinburgh Dental Hospital (or college as we would term it) was founded in 1892, has sixty-four students, and is located on Chambers street, and considered a public charity, but has to pay poor and burgh rates and an income tax; the town council gives them $500 yearly to purchase dental appliances for technical education. They took the occasion of Queen Victoria's diamond jubilee to circulate a subscription paper which netted them $350.

Out of 806 patients, to whom anesthetics were administered for extraction, 290 were given chloroform.

Other hospitals are located, viz: Newcastle Dental Hospital, Liverpool Dental Hospital, Birmingham Dental Hospital, Plymouth Dental Hospital, Exeter Dental Hospital, Victoria Dental Hospital, Manchester; Bristol Royal Infirmary Dental Department, Dental Hospital and School of Ireland (Dublin). The latter, with the Royal College of Surgeons Ireland, grants a diploma in dental surgery. For the final examination each professor asks five questions.

The Dental Hospital of London (Leicester Square), of which we present three views: *one of the waiting room with the lady secretary at her desk, and the chief clinician for the day standing near; on the wall is a large board painted black, on which appear the names and amounts of money donated by each person; this is a permanent arrangement, and when new donors appear their names are added to the list. Other views show exterior of building and amphitheater. The hospital is supported by voluntary contributions, and when patients receive an appointment card a portion reads viz: "Patients are reminded that boxes are placed in the Hall and Surgery for contributions towards the support of the Hospital."

This hospital was founded in 1858 and is open daily, Sundays excepted, and at the outside door is an iron box (with a slot) on which the inscription reads, "Dental Hospital of London, Donations." Thus the charitably inclined passer-by does not lack for opportunity to give.

Adjoining the hospital is a vacant lot on which there is a very large sign which reads; "The Dental Hospital is in need of $200,000 and therefore solicits that amount to erect a new building; contributions can be handed to the secretary."

It is a very common thing to solicit money for dental hospitals and colleges, and nobody takes offense, as it has always been the custom, and we find many ladies among the contributors. In case of the above hospital a wealthy lady has promised to almost donate the lot referred to, which in that part of the "World's Metropolis" is worth a small fortune. In some cases bequests are made in wills; in others donations are given out right; in others there are annual subscribers, both among the profession and laity.

Governors and subscribers can issue tickets to the poor under this law of the hospital: "Every poor applicant suffering pain shall have gratuitous assistance, but necessitous persons requiring

*These views, while good and interesting for the reading of the paper, are not material for the full understanding of the text. -Ed. Reg.

special operations shall be admitted only by the recommendation of a Governor. No donor or subscriber will be supplied with dental appliances. In this hospital the British Dental Association and the Odontological Society of Great Britain hold their meetings, and here Charles S. Tomes lectures on anatomy and physiology. They have a student's dental society, as have all the dental hospitals. In all their history we have found the name of only one student from the United States, H. Murray of California.

Donations are not infrequent, and they lately received one of $1,250 from the trustees of Smith's Charity.

Students who enter this hospital do so upon the understanding that it is their intention to obtain the dental diploma of the Royal College of Surgeons, England.

After passing a preliminary science examination, persons must be apprenticed to a registered dentist for three years, then they can register as a dental student; or in place of working and studying with a dentist, they are often apprenticed or indentured to the Dental Hospital for three years, paying $250 yearly. After this period has elapsed they enter both a dental and general hospital, where they remain for two years, paying $125 each year. They now obtain the degree of L.D.S. (Licentiate of Dental Surgery), diploma fee $100, and can begin to practice if desirable, still studying and passing examinations from time to time until they receive the M.R.C.S. (Member Royal College of Surgeons), or after obtaining the L.D.S., instead of practicing they study at a general hospital for two years, at $125 yearly; they can then present for the degree of M.R.C.S.; diploma (examinations) fee $175.

The fees vary in different colleges and hospitals.

Each student must make 200 fillings per year, twenty-five of them in "dead teeth;" also six artificial dentures, two cases of regulation and two pivot teeth, each year.

The minimum charge for an upper or lower denture is $10, and such payment must be made at the time the case is undertaken, through a Governor, donor, or subscriber to the hospital. The patient must present a special application certified by a clergyman, minister, or justice of the peace, that the applicant is in necessitous circumstances.

In the list of instruments the student is required to have we find the forms of several Americans, viz : Tees, Abbott, Howe, Corydon Palmer, C. R. Butler, Webb, Varney, Donaldson, Weston, Arrington and Taft.

The first *special* dental school established in the United Kingdom was the Metropolitan School of Dental Science, London, 1859. Not in existence at present.

The National Dental Hospital and College, London, is located on the corner of Great Portland and Devonshire streets. The hospital was founded in 1861 and the college in 1877. They occupy all of a new three-story building, 50 x 60 feet, finished interiorly with hardwood and white enameled brick, thus adding to the light and sanitary condition. The rooms are well furnished and equipped.

His Royal Highness the Duke of York, K.G., is president of the hospital, and among the managers are many other titled gentlemen.

The consulting dental surgeon is Sir Edwin Saunders, F.R.C.S. He is also surgeon-dentist to Queen Victoria.

The lecturer on operative dental surgery is George Cunningham, D.M.D., L.D.S., who wrote the prize essay on Dental Hygiene for the Columbian Dental Congress in 1893. One of the dental surgeons is Thomas G. Read, D.M.D., Harvard, L.D.S., England, and one of the graduates is Charles M. Cunningham, D.D.S., Michigan.

Students must enter for two years and attend two-thirds of all lectures before they are entitled to examination for a degree. Lectures begin in October and end in July, and some of them are given at 5, 6 and 7 P. M. Ladies are admitted as students both to the college and hospital. The stopping rooms (operating rooms) are lighted by electricity and have accommodation for sixty chairs. The college provides all the special dental lectures required by the curriculum for the dental diploma of the Royal College of Surgeons, England, and it pays $500 per year for poor rates and taxes.

They have life governors, annual governors, life subscribers and annual subscribers. Every donor to the hospital of $100 shall be a life governor, and entitled to recommend twenty patients annually. Every donor of $15 annually shall be an annual governor, and entitled to the same privileges as life governors. Every donor of $50 shall be a life subscriber, and entitled to recommend ten patients annually. Every donor of $5 annually shall be an annual subscriber, and entitled to recommend five patients annually. Every donor of $10 annually is entitled to recommend ten patients annually. Every donor of $25 is entitled to recommend five patients annually. Every executor, under direction in the will of a testator, who pays $500 or more, shall be a life governor. Every clergyman who, by a collection in his church obtains and donates $25, shall be entitled to recommend ten patients during that year; if he donates $100 he shall be an honorary governor for five years, and if he donates $200 he shall be an honorary governor for life.

In the annual announcement is published a list of donors and subscribers, with the amount opposite each name. The names of seventy ladies (and more gentlemen) appear in the last list, and the amounts vary from $5 to $2,000. Baroness Burdett Coutts is one of the contributors; also the corporation of the city of London.

Before a person can register as a student he must pass an examination, viz: (1) English grammar and composition. (2) Latin grammar and translations of easy passages. (3) Arithmetic, including fractions, algebra, simple equations, geometry, 1-2-3 books of Euclid. (4) Elementary mechanics of solids and fluids, elements of statics, dynamics, hydrostatics. (5) One of the following subjects: Greek, French, German, Italian, logic.

Among the list of instruments the student is required to have some are of American manufacture and others are copies. A very few of the text-books are of American authorship.

A medical education entitles its possessor to practice dentistry, yet the course of study for such does not include any special dental instruction.

Thomas Guy, at whose "sole costs and charges" Guy's Hospital was founded, was born in 1645, at Horselydown. At the age of 15 he was apprenticed to John Clark, bookseller. When his apprenticeship ended he became a freeman of the Stationers' Company of London, and started in business with $1,000. He printed a large number of Bibles, having obtained from the University of Oxford an assignment of their privilege. He sat in all parliaments from the third of William III. to the first of Queen Anne. He leased ground for 999 years, and he erected the first building which was dedicated in 1722. He died in 1724, aged eighty.

In 1829 William Hunt, of London, died and left a bequest to the hospital of $900,000. A lady has recently given $30,000 and a gentleman $25,000. The endowment fund now exceeds $1,000,000.

The Rt. Hon. W. E. Gladstone was a staunch friend, and the hospital proposes to erect a memorial to him—to be placed in the hospital.

Guy's Hospital Dental School, near London bridge, was opened in 1889, and in 1890 a well-lighted operating room (conservation room) large enough for fifty Morrison chairs, and a spacious laboratory and waiting rooms were built. At this hospital there are sixty sets of rooms where students can be accommodated with room and board at a moderate charge, but if they enter the building after 11 P. M., they are reported. The students' club contains reading rooms, library, gymnasium, and dining-hall, where dental and medical student members are supplied with luncheons, dinners, etc.; they also have a nine-acre field near the city, where the Clubs' Union play cricket, foot ball, tennis, target practice with the rifle, and ride the bicycle. The library referred to has 6,500 volumes, which are accessible on making a deposit of five dollars.

The preliminary examination for entering the dental or medical school is the same, and is very like a schedule already given. Every inducement to take the M.R.C.S. and L.R.C.P., together with the L.D.S., is offered by studentship at this hospital; patients are not required to obtain cards of admission as in the case of hospitals already noticed. Patients not entitled to free fillings, pay twelve cents each for amalgam or cement, and twenty-five cents for gold not exceeding sixteen grains, any excess is at rate of eight cents per grain. The charge for crowns is from twenty-five to seventy-five cents, and for bridge-work seventy-five cents per tooth. No charge for artificial dentures or regulation plates.

The winter session begins October 1st and ends March 31st. Summer session begins May 1st and ends July 31st; the course lasts through two years, exclusive of apprenticeship. Students before graduating must make three hundred and fifty fillings; sixteen crowns; six regulation cases; six dentures; twenty scalings; treat forty pulpless teeth.

During the past year twenty-eight thousand patients received attention.

Among their text-books we noticed Essig's Prosthetic Dentistry and Richardson's Mechanical Dentistry, and among the forms of American instruments—Evans, Abbott, Howe, Case, Butler, Varney, Webb, Donaldson, Ivory.

The English dental hospitals do not give a list of members in each class, but do give a list of graduates since the founding.

On the surgical and medical staff of Guy's Hospital there are three dental surgeons, and accommodations are furnished for dental patients in one of the wards.

[TO BE CONTINUED.]

